# Omicron: a drug developer’s perspective

**DOI:** 10.1080/22221751.2021.2023330

**Published:** 2022-01-04

**Authors:** Fang “Flora” Fang, Pei-Yong Shi

**Affiliations:** a Abimmune Biopharma, Inc., San Diego, CA, USA; b Department of Biochemistry and Molecular Biology, Department of Biochemistry and Molecular Biology, The University of Texas Medical Branch, Galveston, TX, USA

**Keywords:** COVID19, SARS-CoV-2, Omicron variants, COVID19vaccines, COVID19drugs

## Abstract

We performed an annotation of 35 mutations in the spike protein of the SARS-CoV-2 Omicron variant. Our analysis of the mutations indicates that Omicron has gained prominent immune evasion and potential for enhanced transmissibility. Previous modeling study has revealed that continued evolution in both immune evasion and enhanced transmissibility by SARS-CoV-2 would compromise vaccines as tools for the pandemic control. To combat the future variants of SARS-CoV-2, the world needs novel antiviral drugs that are effective at curb viral spreading without introducing additional selective pressure towards resistant variants.

Since its first detection on 16 August 2021 in South Africa, Omicron has now spread to 69 countries in the world, including 42 states in the USA (outbreak.info). Carrying up to 61 nonsynonymous defining mutations, Omicron, also known as B.1.1.529 or variant 21K or BA.1, has opened our eyes on the extent to which SARS-CoV-2 can evolve. Recently, a sister variant of Omicron, known as 21L or BA.2, has also been discovered. (https://covariants.org/variants/21K.Omicron) Due to its unusually high number of mutations, projected enhancement in immune evasion and transmissibility, Omicron was designated as a new variant of concern (VOC) by the WHO on 26 November 2021.

On a global scale, positive selection of SARS-CoV-2 mutations appears to have begun in late 2020 [1]. Since then, the virus has been evolving on two fronts: immune evasion and enhanced transmissibility, as demonstrated by Delta. This evolutionary mode is the most conducive for viral spreading and a long-lasting epidemic [2]. In the past year, the world has been confronted with a slew of ever more powerful variants, and now, Omicron has come. This raises an obvious question: how bad is Omicron?

To shed some lights on this question, we have annotated the 35 Omicron spike mutations (Table 1). This exercise has given us some insights on what Omicron might be capable of. Additionally, it also brings a new perspective on future approaches in combating SARS-CoV-2.

**Table 1.** Annotation of the Omicron spike mutations.

Omicron (B.1.1.529) spike mutations		Annotation	Mutation presence & prevalence up to 30 November 2021	Mutation impact
Spike protein N-terminal domain (NTD)	A67V	Evaded nAb binding [4].	Eta	Immune evasion
	ΔH69-V70	In the N2 neutralizing epitope and assiciated with convalescent plasma escape. Evaded nAb binding [4].	Alpha, Eta & C.36.3-related	Immune evasion
	T95I	Alters the N3 epitope topography.	B.1.1.318-related & Iota	Immune evasion
	G142D, Δ143-145	In the N3 neutralizing epitope. Abolished binding by nAbs [3,4].	Alpha, Eta & C.1.2.; Mu (Y144S, Y145N)	Immune evasion
	Δ211/L212I, ins214EPE	In a predicted HLA class II epitope [5].		Immune evasion?
Spike receptor binding domain (RBD) & Receptor binding module (RBM)	G339D	In the classes 3 & 4 neutralizing epitope [6].	Detected in 469 sequences	Immune evasion?
	S371L, S373P, S375F	In the classes 3 & 4 neutralizing epitope [6].	S:371L: in 11 sequences. S373P: in 322 sequences. S375F: in 208 sequences.	Immune evasine?
	K417N	In the class 1 neutralizing epitope and evaded nAbs [6,7].	Beta; Gamma (K417T). Delta (AY.2)	Evasion of both humoral & cellular immunity
	N440K	In the class 3 neutralizing epitope and may escape nAbs [6,7].	Detected in 8,153 sequences	Immune evasion
	G446S	In the class 3 neutralizing epitope [5,6]. May evade some nAbs [7].	Detected in 671 sequences	Immune evasion
	S477N	In the class 1 neutralizing epitope [6]. Strongly resistant to a panel of nAbs [19].	Iota; Detected in 70,964 sequences	Immune evasion
	T478K	May evade some nAbs [19].	Delta.	?
	E484A	In the class 2 neutralizing epitope [7]. Strongly resistant to a panel of nAbs [7]. Multiple E484 mutations conferred resistance to convalescent sera [19].	E484K: Beta, Gamma, Iota, Mu, Eta, & Theta; E484Q: Kappa	Immune evasion
	Q493R	In the class 2 neutralizing epitope and may escape nAbs.	Detected in 145 sequences	Immune evasion
	G496S	May be partially resistant to nAbs.	Detected in 480 sequences	?
	Q498R	At ACE2 interphase. May increase ACE2 affinity to low pM-level when combined with N501Y, E484K, & S477N [13].	Detected in 113 sequences, 22 sequences carry Q498R & N501Y dual mutations.	Enhanced infectivity & Immune evasion
	N501Y	At ACE2 interphase; In class 1 neutralizing epitope and may evade some nAbs.	Alpha, Beta, Gamma, Mu	Enhanced infectivity & Immune evasion
	Y505H	In the class 1 neutralizing epitope.	Detected in 130 sequences	Immune evasion
Spike protein C-terminal segment	T547K		Detected in 607 sequences	?
	D614G	Decreased HLA class I binding. Increases infectivity and transmissibility [20].	Alpha, Beta, Gamma, Delta, Kappa, Lambda, Eta, Iota, Mu, Theta, C.1.2, B.1.1.318, & C36.3	Enhanced infectivity & Evasion of both humoral & cellular immunity
	H655Y	Adjacent to the S1/S2 furin cleavage site	Gamma, C.1.2	Enhanced infectivity
	N679K	Adjacent to the S1/S2 furin cleavage site	C.1.2	Enhanced infectivity
	P681H	Adjacent to the S1/S2 furin cleavage site & in a HLA-B7 epitope [20]. Type I IFN resistance.	Alpha, Mu; P681R: Delta, Kappa	Enhanced infectivity. Evasion of innate & cellular immunity
	N764K		Detected in 345 sequences	?
	D796Y	In an HLA-II epitope [4]. In a epitope recognized by convalescent sera [11].	Detected in 4053 sequences	Immune evasion?
	N856K	Adjacent to the fusion-peptide triad (K835, Y837 & K854) that interact with D614.	Detected in 57 sequences	Altered infectivity?
	Q954H	In a predicted HLA class II epitope [5].	Detected in 30 sequences	Immune evasion?
	N969K	In a predicted HLA class II epitope [5].	Detected in 63 sequences	Immune evasion?
	L981F		Detected in 40 sequences	?

Omicron appears to have learned from the older variants. Only six of its 61 mutations are unique, the rest already existed in the sequenced genomic pool of SARS-CoV-2, including 20 very abundant convergent mutations that Omicron shares with other prominent variants. Notably, the Omicron spike protein contains 15 convergent mutations, all of which confer an advantage either in immune evasion or in transmissibility (Table 1).

In the N-terminal domain (NTD) of the SARS-CoV-2 spike protein, neutralizing epitopes have been mapped to the N2 and N3 loops [3]. The N-terminal eight mutations in the Omicron NTD have altered these epitopes. These mutations also exist in previous variants and have been shown to cause evasion of the NTD-targeting neutralizing antibodies (nAbs) [4]. Furthermore, in the Omicron NTD, a combination of mutations (Δ211/L212I, ins214EPE) that are unique to Omicron may disrupt an HLA class II epitope, resulting in immune evasion through impaired dendritic cell priming [5]. Thus, the NTD in Omicron appears to have become insensitive to the existing nAbs targeting this region.

Most existing nAbs targeting the receptor binding domain (RBD) of the spike protein have been mapped to fout areas in RBD and classified as classes 1–4 [6,7]. All epitopes from the four classes contain mutations, with 15 total in Omicron. The number of mutations in each class ranges from 2 to 4. Most of the mutations have also been previously shown to impair nAbs binding, suggesting that Omicron may be resistant to the existing nAbs of classes 1–4.

Consistently, of the eight approved or authorized antibodies, all but sotrovimab has lost their neutralizing activity against the Omicron pseudovirus [8]. Sotrovimab binds to a cryptic RBD epitope [9] that seems to be less affected in Omicron, thus its neutralizing potency against Omicron only dropped by three-fold [8]. Similarly, neutralizing efficacy of convalescent sera were reduced against Omicron psedovirus by 8.4-folds on average [10].

Despite the bleak projections derived from Omicron’s NTD and RBD, analysis of the spike C-terminal segment gave us reasons for optimism. For example, the most recognized neutralizing epitopes by the vaccinated and convalescent sera were mapped to three sites (aa 558-569, 627-638, and 1148-1159) in the C-terminal segment of the spike protein [11]. These sites remain intact in Omicron. Thus, one can hope that the current vaccines remain effective against Omicron.

Recent results, however, indicate that Omicron significantly escapes two-doses’ vaccines, ranging from complete loss to 33- to 44-fold reduction of neutralizing activities [8,12] Sera from people who received the third dose of vaccines maintained about 10% of the neutralizing activity, and such neutralizing activity was completely lost after three months [12].

These findings may temper the prospect of controlling spread of Omicron and the future SARS-CoV-2 variants by vaccination, especially if the virus continues to enhance its abilities in immune evasion and transmissibility. On 20 December 2021, the CDC estimated that Omicron may have 1.6-fold increased transmissibility compared to Delta (https://www.cdc.gov/coronavirus/2019-ncov/science/forecasting/mathematical-modeling-outbreak.html).

Omicron carries three sets of unique changes that may enhance its transmissibility through different mechanisms: the Q498R and N501Y dual mutations that enhance ACE2 binding [13], the H655Y, N679K, and P681H triple mutations that could contribute to enhanced cleavage at the S1/S2 junction, and the R203K and G204R dual mutations in N protein that increased viral load in patients [14]. Particularly disconcerting are the Q498R and N501Y dual mutations, which, when combined with E484K and S477N mutations could increase the affinity to ACE2 by up to 1000-fold and up to the level of low pM in K_D_ value [13]. During infection, a virion binds to ACE2 in a multivalent mode, thus its binding avidity could be further enhanced by orders of magnitude, even to the level of fM in K_D_ value.

Such a high affinity to the receptor, if indeed acquired by Omicron or its future derivatives, means that vaccines and antibody-based therapies may lose their efficacy in curtailing SARS-CoV-2, even if we keep them constantly updated against new variants. This is because it would be challenging for even the best antibodies, with affinities in the nM- or even pM-level in K_D_ values, to compete against the virus. For the same reason, vaccines that function though inducing nAbs would be compromised in their potencies.

Fortunately, in addition to the affinity enhancing mutations, Omicron also carries several mutations that may reduce its affinity to ACE2 as shown by deep mutational scanning, including K417N, G446S, E484A, Q493R, G496S, and Y505H [15]. As a result, Omicron’s receptor binding domain (RBD) only has a modest 2.4-fold increased binding affinity to human ACE2 [8]. Although this finding may bring a sigh of relief at this time, mutations that may significantly elevate viral receptor binding affinity can still happen in the future and need to be closely monitored.

Receptor binding affinity is only one of the many aspects that may impact the transmissibility of a virus. Although most research so far has focused on mutations in the spike protein, mutations outside of the spike protein may also play important roles in viral immune evasion and transmissibility [16]. More studies are needed to address this knowledge gap. It is also unclear if Omicron causes more severe disease as current data are conflicting and inconclusive. The good news is that despite the significant number of mutations that Omicron carries, most T cell epitopes seems to be preserved in Omicron, which indicates that through CD8+ T cell responses, vaccines may remain protective against severe COVID19 caused by Omicron [17].

Omicron has revealed to us that SARS-CoV-2 has the potential to go beyond the protective threshold provided by vaccines and antibodies. Playing catchup to SARS-CoV-2 selects for more resistant and transmissible variants and may not be successful in the long run. Besides updating vaccines against Omicron or its future derivatives, we need to speed up the development of novel antivirals.

So, which therapeutic properties are most impactful against COVID19? A recent modelling study has pointed out that while reducing COVID19 severity, mortality, or preventing hospitalization are all beneficial, reducing the duration of infectiousness would provide three-times the beneficial impact of any of the other properties [18].

A future COVID19 therapy should ideally meet three criteria: (1) it should have high potency and resistance barrier; (2) it should be effective at reducing viral replication and minimize viral spreading; and (3) it should be effective against all SARS-CoV-2 variants, including the future ones, and avoid introducing additional selective pressure towards resistant variants.

Is such a therapy possible? Numerous scientists are working hard towards that goal, and our pipeline of innovative therapies is growing by the day. Novel antiviral drugs, such as Molnupiravir (MK-4482) and Paxlovid^TM^ (PF-07321332), have already demonstrated promising efficacy in clinical trials and are poised to enter the market on an emergency use basis (https://www.bloomberg.com/news/articles/2021-12-21/fda-expected-to-authorize-pfizer-merck-covid-pills-this-week?srnd=premium). Representing an alternative platform of host-targeting drugs with a reduced risk of selecting resistant variants, a candidate being developed by Abimmune Biopharma, named *ResCovidin^TM^*, has been designed to block all ports of cell entry used by the virus with a potency that may rival Omicron’s level of infectiousness (unpublished results).

Fighting SARS-CoV-2 is the great battle of our time. Working together, we shall prevail.
